# Tumor-derived exosomal microRNA-7-5p enhanced by verbascoside inhibits biological behaviors of glioblastoma *in vitro* and *in vivo*

**DOI:** 10.1016/j.omto.2020.12.006

**Published:** 2020-12-19

**Authors:** Huan Wang, Jiugeng Feng, Fan Ao, Yiqiang Tang, Pengliang Xu, Min Wang, Min Huang

**Affiliations:** 1 Department of Neurosurgery, Jiangxi Provincial Corps Hospital of Chinese People’s Armed Police Forces, Nanchang 330001, PR China; 2 Department of Neurosurgery, The First Affiliated Hospital of Nanchang University, Nanchang 330006, PR China; 3 Department of Radiation Oncology, Jiangxi Cancer Hospital, Nanchang 330029, PR China

**Keywords:** verbascoside, glioblastoma, miR-7-5p, tumor-derived exosomes, proliferation, metastasis, microtubule formation, EGFR/PI3K/Akt signaling pathway

## Abstract

Verbascoside (VB), a glycosylated phenylpropane compound, has been widely used in traditional medicine showing anti-inflammatory and anti-tumor effects in many diseases. The current study aimed to investigate the mechanism underlying the inhibitor effect of VB on glioblastoma (GBM). We isolated and identified the tumor-derived exosomes (TEXs) secreted by GBM cells before and after treatment with VB, after which, we detected expression of microRNA (miR)-7-5p in cells and TEXs by qRT-PCR. Loss- and gain-function assays were conducted to determine the role of miR-7-5p in GBM cells with the proliferation, apoptosis, invasion, migration, and microtubule formation of GBM cells detected. A subcutaneous tumor model and tumor metastasis model of nude mice were established to validate the *in vitro* findings. We found that VB promoted the expression of miR-7-5p in GBM and transferred miR-7-5p to recipient GBM cells by exosomal delivery. Consequently, miR-7-5p downregulated epidermal growth factor receptor (EGFR) expression to inactivate the phosphatidylinositol 3-kinase (PI3K)/protein kinase B (Akt) signaling pathway, causing inhibition in the proliferation, migration, invasion, and microtubule formation of GBM cells *in vitro*, as well as decline in tumor formation and metastasis *in vivo*. Overall, VB can promote the expression of miR-7-5p in GBM cells and transfer miR-7-5p via exosomes, thereby inhibiting the occurrence of GBM.

## Graphical Abstract

**Figure:**
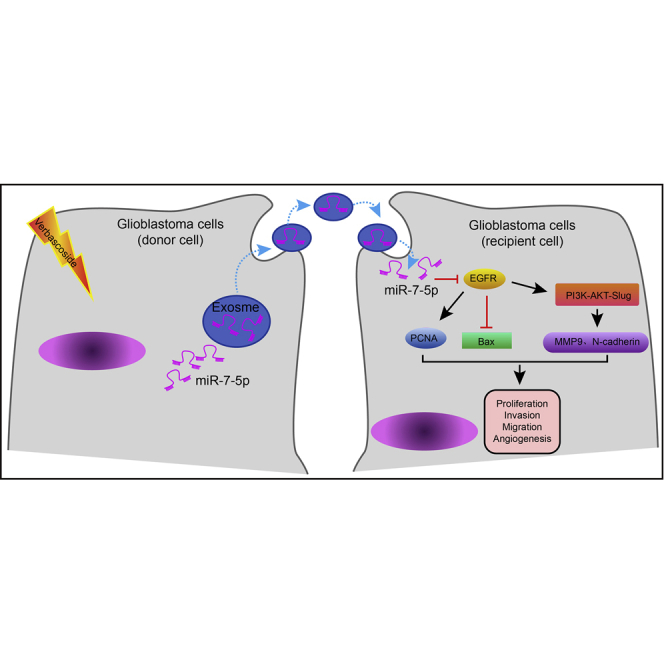

## Teaser

VB elevates the expression of miR-7-5p in GBM cells and transferred miR-7-5p by exosome secretion to recipient cells, causing an inhibition of proliferation, migration, invasion, and angiogenesis *in vitro* and tumor formation and metastasis *in vivo*, thus delaying the progression of GBM.

## Introduction

Glioblastoma (GBM) is the most malignant form of glioma in astrocytoma, with invasive characteristics often invading several brain lobes and invading deep structures.^1^ Statistics demonstrated that the median survival of patients with GBM was limited to 16−19 months and only about 25% to 30% of the patients alive at 2 years after diagnosis.^2^ Notably, GBM, due to its underlying tolerance to radiotherapy, elicits variable sensitivity rates ranging from 40% to 80% depending on the chemotherapeutic protocol.^3^ Clinically, the therapeutic effect of GBM has not been ideal, and hence, it necessitates extensive investigation to find a more effective treatment. Recently, several therapeutic drugs targeting the key targets of tumorigenesis have been advantageous, so it is principally important to study the underlying mechanism of GBM.^2^^,^^4^

Verbascoside (VB), also known as acteoside, is a phenylethanoid glycoside that was first isolated from mullein (Verbascum sinuatum L., Scrophulariaceae) but is also found in several other plant species, such as Plantago psyllium L.^5^ VB is hydrophilic in nature and has prominent pharmacologically beneficial activities for human health, including antioxidant, antimicrobial, anti-inflammatory, neuroprotective, and wound-healing effects, along with antiproliferative effects in cancer.^6^ VB was previously recognized for its potent anti-proliferative, pro-apoptotic, and pro-differentiative chemo-preventive/chemotherapeutic potentials.^7^ Its anti-cancer effect and property have been ascertained in a variety of tumors, including GBM; however its specific mechanism of action to inhibit GBM has not been studied.^8^ Existing evidence has shown the ability of VB to suppress the growth of GBM cells through repression of CD44 dimerization.^9^ Furthermore, VB can explicitly inhibit the proliferation, migration, and invasion of GBM cells by upregulating the protein tyrosine phosphatase src homology 2 domain-containing protein tyrosine phosphatase 1 (SHP-1) and inhibiting the phosphorylation of signal transducer and activator of transcription 3 (STAT3).^8^

Exosomes are small, single-membraned vesicles approximately 30−200 nm in diameter, which have the same topology as cells and are enriched with plenty of proteins, lipids, nucleic acids, and glycoconjugates.^10^ Current studies have elucidated tumor-derived exosomes (TEXs) as a key component of carcinogenesis, development, and cancer treatment, where exosomes are also involved in the transmission of microRNA (miRNA or miR) to specific cells.^11^, ^12^, ^13^, ^14^ Some researchers documented that exosomes derived from tumor cells can promote proliferation of glioma cell line. Moreover, they also detected various miRNAs, a class of noncoding RNAs capable of targeting specific messenger RNAs (mRNAs), implicated in various biological processes,^15^ in serum exosomes of GBM patients.^16^ Existing evidence has shown that miR-7-5p plays an inhibitory role in the development of GBM.^17^ Additionally, miR-7-5p has been reported to regulate the epidermal growth factor receptor (EGFR) and EGFR/phosphatidylinositol 3-kinase/protein kinase B/mechanistic target of rapamycin (PI3K/Akt/mTOR) signaling pathway to affect tumor cell growth.^18^^,^^19^ It is reported that the EGFR/PI3K/Akt/mTOR signaling pathway and inhibition of STAT3 may be the imperative mechanisms mediating oxymatrine-regulated anti-tumor action of GBM cells.^19^ At present, the research regarding the inhibition of VB on GBM extracellular miRNA in GBM is not sufficient. In this study, VB can affect the expression of miR-7-5p in GBM and induce the release of miR-7-5p via exosomes. Additionally, exosomal miR-7-5p could suppress the malignant phenotypes of GBM cells through inhibition of the EGFR/PI3K/Akt signaling pathway.

## Results

### VB inhibits the proliferation, migration, and invasion while promoting the apoptosis of GBM cells

**Figure 1:**
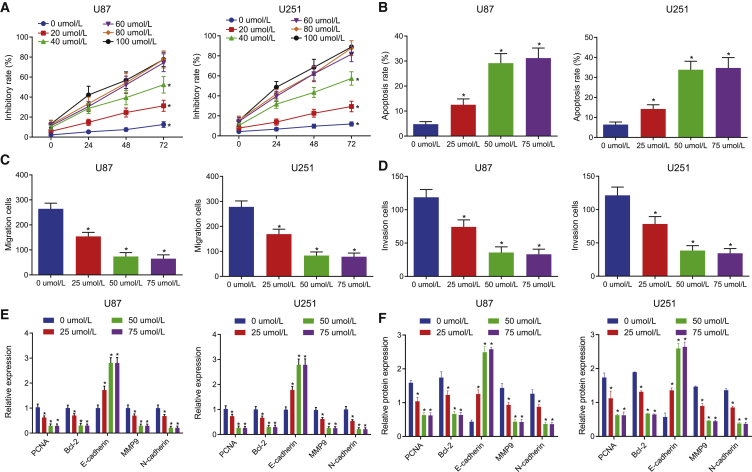
VB inhibits proliferation, migration, and invasion but promotes apoptosis of GBM cells (A) The proliferation of U87 and U251 cell lines treated with VB at different concentrations (0, 20, 40, 60, 80, and 100 μΜ) at 0, 24, 48, and 72 h was detected by MTT assay; U87 and U251 cell lines were treated with VB at different concentrations (0, 25, 50, and 75 μΜ). (B) The apoptosis of U87 and U251 cell lines was measured by flow cytometry. (C) The cell migration of U87 and U251 cell lines was measured by Transwell assay. (D) The cell invasion of U87 and U251 cell lines was measured by Transwell assay. (E) The mRNA expression of PCNA, Bcl-2, E-cadherin, MMP9, and N-cadherin was detected by qRT-PCR in U87 and U251 cell lines. (F) The protein expression of PCNA, Bcl-2, E-cadherin, MMP9, and N-cadherin was detected by western blot analysis in U87 and U251 cell lines. Data were shown as mean ± standard deviation of three technical replicates. ANOVA of repeated measurement was used for data comparison in (A) and one-way ANOVA for data comparison in (B−E) with Tukey’s post hoc test. ∗p < 0.05, compared with the previous drug concentration at the same time point; #p < 0.05, compared with the previous time point at the same drug concentration.

We chose U87 and U251 cell lines as the research objects to explore the effect of VB on the biological characteristics of GBM cells. First, we used 3-(4,5-dimethylthiazol-2-yl)-2, 5-diphenyltetrazolium bromide (MTT) to detect the proliferation activity of GBM cells treated with different concentrations of VB (0, 20, 40, 60, 80, and 100 μΜ) at different time points (0, 24, 48, and 72 h). Our results showed that the proliferation activity of U87 and U251 cells was decreased with the increase of the concentration of VB in a certain range (p < 0.05) (Figure 1A), indicating that VB could inhibit the proliferation of GBM cells. In addition, it is worth noting that upon treatment with VB at concentrations of 60, 80, and 100 μM, there was no significant difference in cell proliferation activity (p > 0.05), which suggested that the optimal concentration of VB was between 40 μΜ and 60 μΜ for our experiment.

Next, we used flow cytometry to detect the apoptosis of U87 and U251 cell lines under the effect of VB (0, 25, 50, and 75 μΜ) for 48 h. We found that the apoptosis rate of GBM cells was increased significantly with the increase of drug concentration under the effect of VB (0−50 μΜ), and the difference was statistically significant (p < 0.05). By contrast, no significant difference was evident in the apoptosis rate of cells treated with VB at concentrations of 75 μΜ and 50 μΜ (p > 0.05) (Figure 1B).

For the reason that the proliferation ability of cells in each group was similar within 24 h, we observed the effect of different concentrations of VB on the migration and invasion of U87 and U251 cells at 24 h to reduce the error. Transwell assay showed that the migration and invasion ability of GBM cells decreased significantly with an increase in VB concentration in the range of 50 μΜ (p < 0.05) (Figures 1C and 1D).

Meanwhile, the results of quantitative reverse transcriptase polymerase chain reaction (qRT-PCR) and western blot analysis revealed that expression of E-cadherin was increased, whereas that of proliferating cell nuclear antigen (PCNA), B cell lymphoma 2 (Bcl-2), matrix metalloproteinase 9 (MMP9), and N-cadherin was reduced in cells treated with 0−50 μΜ of VB (p < 0.05) (Figures 1E and 1F). These results were consistent with the observed effect of VB on the biological characteristics of GBM cells. Therefore, it might be plausible to suggest that VB could inhibit the proliferation, migration, and invasion and promote apoptosis of GBM cells. In the following experiment, we selected VB at the concentration of 50 μΜ as the optimal working concentration.

### Microtubule formation can be inhibited by the supernatant of GBM cells treated with VB instead of VB itself

**Figure 2:**
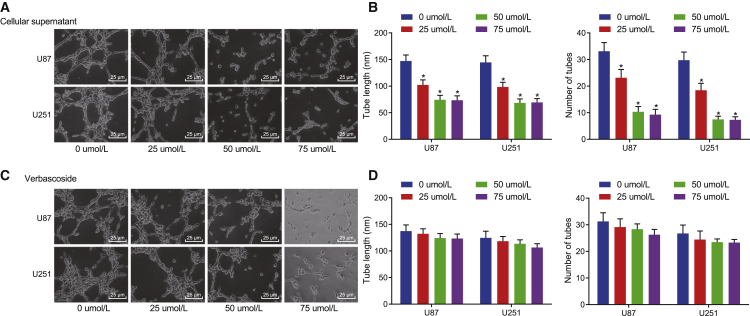
The supernatant of GBM cells treated with VB inhibits microtubule formation (A) The effect of supernatant of U87 and U251 cells on microtubule formation after 48 h of treatment with different concentrations of VB (×400). (B) Tube length and the number of tubes are quantified for (A). (C) The effect of different concentrations of VB on microtubule formation of GBM cells (×400). (D) Tube length and the number of tubes are quantified for (C). Data were shown as mean ± standard deviation of three technical replicates. One-way ANOVA was used for data comparison in panel B and D, with Tukey’s post-test test. ∗p < 0.05, compared with the previous drug concentration.

The following experiments focused on exploring whether VB can inhibit the microtubule formation of GBM cells. We observed the growth of human umbilical vein endothelial cells (HUVECs), which were co-cultured, respectively, with the supernatant of U87 and U251 cells treated with VB of variable concentrations (0, 25, 50, and 75 μΜ) for 48 h. Our results showed that the microtubule formation ability of GBM cells was decreased with an increase in VB concentration (0−50 μΜ), with the difference statistically significant (p < 0.05), whereas there were no significant changes of the microtubule formation ability of GBM cells treated with VB at the concentrations of 75 μΜ and 50 μΜ (p > 0.05) (Figures 2A and 2B).

But, as the aforementioned was insufficient to prove that VB can inhibit the microtubule formation, it was uncertain whether VB had a direct effect on HUVECs. So, we cultured HUVECs with the supernatant of GBM cells without VB treatment to eliminate this interference, and we simultaneously supplied the corresponding concentration of VB. Our results showed that the microtubule formation of HUVECs did not change significantly upon addition of supernatant supplemented with different concentrations of VB to the HUVECs compared with the previous concentration (p > 0.05) (Figures 2C and 2D). These results indicated that the inhibition of microtubule formation previously observed (Figures 2A and 2B) was not the direct effect of VB on HUVECs.

All of the results indicated that the supernatant of GBM cells treated with VB can inhibit the microtubule formation instead of acting on HUVECs directly by VB, which speculated that it was possible that the microenvironment of GBM cells had changed after the effect of VB.

### GBM-derived exosomes contain miR-7-5p

**Figure 3:**
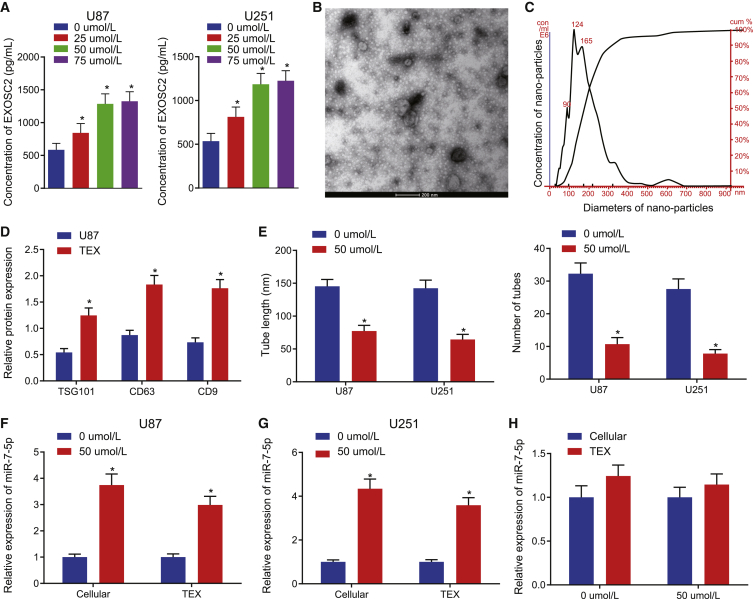
GBM cell-derived exosomes encapsulate miR-7-5p (A) Content of EXOSC2 was detected in the supernatant of GBM cells treated with VB at different concentrations (0, 25, 50, and 75 μΜ) for 48 h. (B) The morphology of exosomes in the supernatant of GBM cells treated with VB was observed under a transmission electron microscope (scale bar, 200 nm). (C) The size distribution of TEXs was detected by Zetasizer Nano ZS in GBM. (D) The expression of exosome surface marker proteins was detected by western blot analysis in the TEXs. (E) The effect of purified TEXs in the supernatant of GBM cells treated with VB for 48 h on the ability of microtubule formation was examined. (F) The expression of miR-7-5p in U87 cells and purified TEXs under the effect of VB was detected by qRT-PCR. (G) The expression of miR-7-5p in U251 cells and purified TEXs under the action of VB was detected by qRT-PCR. (H) The expression of miR-7-5p in GBM cells and TEXs under the action of VB was detected, respectively, by qRT-PCR. Data were shown as mean ± standard deviation of three technical replicates. One-way ANOVA was used for data comparison in (A) with Tukey’s post-test test. Unpaired t test was used for data comparison in (E−H). ∗p < 0.05, compared with the previous drug concentration or 0 μΜ group.

In recent years, several studies have flagged that tumor cells can produce exosomes that involve multiple cell functions and participate in certain physiological and pathological events.^11^, ^12^, ^13^, ^14^ These tumor-sourced exosomes affect the surrounding environment and form a communication network *in vivo* through local metastasis information and systemic metastasis to distant tissues, then promote tumor progression and metastasis, and can also cause an anti-tumor reaction at times. So, in order to verify the hypothesis that VB changed the microenvironment of tumor cells after acting on GBM cells, we detected the assay of EXCSC2 in the supernatant of U87 and U251 cells treated with VB at different concentrations to reflect the condition of cancer cells secreting exosomes. Our results showed that the concentration of exosomes in the supernatant of GBM cells was increased significantly with an increase in VB concentration (0−50 μΜ; p < 0.05) (Figure 3A), which indicates that VB can promote the secretion of exosomes in GBM cells. Next, we isolated exosomes from the U87 cell line and identified the characteristics of the TEXs. First, a group of round or oval membrane vesicles with a diameter of 40−150 nm and basically the same shape can be seen under a transmission electron microscope (TEM), and its membrane structure can be seen around the periphery of the vesicles, with low electron density components in the center (Figure 3B). The diameter of TEXs was observed to be 64.38 nm by Zetasizer Nano ZS (Figure 3C). Western blot analysis results indicated that the expression of exosome surface markers TSG101, CD63, and CD9 was much higher in TEXs compared with U87 cells, which suggested the successful exosome extraction (Figure 3D). Then, we purified the TEXs in the supernatant of GBM cells treated with VB (0, 50 μΜ) for 48 h, and we observed the effect of TEXs on the microtubule formation of cancer cells. Our results showed that the TEXs could significantly reduce the microtubule formation of cancer cells after stimulating secretion of GBM cells by VB (p < 0.05) (Figure 3E).

Alternatively, miR-7-5p has been proven to be significantly poorly expressed in the event of GBM.^20^ So, we detected the expression of miR-7-5p in the U87 and U251 cells under the effect of VB with qRT-PCR. The results showed that the expression of miR-7-5p was significantly higher in the GBM cells with VB than that in the control cells. Additionally, in the purified TEXs, the expression of miR-7-5p was also found to be significantly higher upon the addition of VB than that in the control TEXs (p < 0.05) (Figures 3F and 3G). These results suggested that the exosomes from GBM contained miR-7-5p, and this process could be promoted by VB. Interestingly, there was no significant difference (p > 0.05) in the expression of miR-7-5p in cells following treatment with 50 μΜ of VB compared with that in TEXs (Figure 3H), which suggested that the exosomes secreted from GBM stimulated by VB may be transferred to the recipient cells.

### VB promotes the expression of miR-7-5p in GBM and facilitates its delivery via exosomes to recipient GBM cells

**Figure 4:**
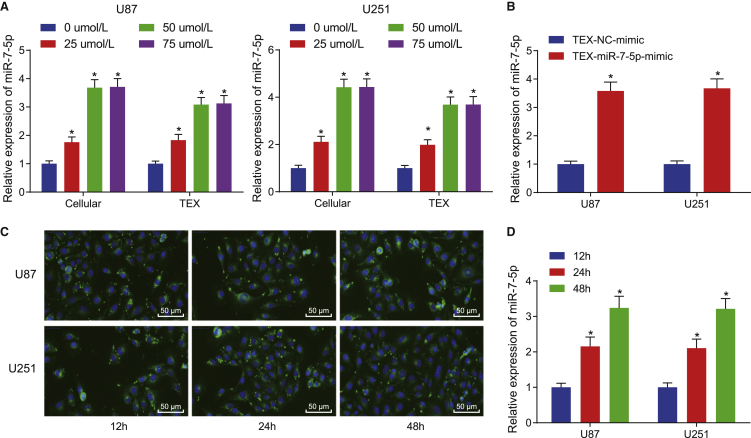
VB promotes the expression of miR-7-5p in GBM and boosts its transfer by exosomes to recipient GBM cells (A) The expression of miR-7-5p in the cell and in the supernatant TEXs from the GBM cells treated with VB at different concentrations (0, 25, 50, and 75 μΜ) for 48 h was detected by qRT-PCR. (B) The expression of miR-7-5p in purified TEXs from miR-7-5p-mimic-transfected U87 and U251 cells was detected by qRT-PCR. (C) The uptake of exosomes by GBM cells was observed under a fluorescence microscope after tracing with CFSE (×200). (D) The expression of miR-7-5p was detected by qRT-PCR in U87 and U251 cells at 12, 24, and 48 h. Data were shown as mean ± standard deviation of three technical replicates. One-way ANOVA was used for data comparison in (A) and (D) with Tukey’s post-test test. Unpaired t test was used for data comparison in (B). *∗*p < 0.05, ∗∗p < 0.01, compared with 0 μΜ group, TEX-NC-mimic group, or 12 h group.

First, we detected the expression of miR-7-5p in U87 and U251 cell lines following VB treatment for 48 h with qRT-PCR to confirm the effect of VB on the expression of miR-7-5p in the GBM cells. Expectedly, the expression of miR-7-5p was increased significantly in GBM cells and in the upper-serum TEXs with an increase in VB concentration (0−50 μΜ; p < 0.05) (Figure 4A), and the expression of miR-7-5p was also found to be abundant in the TEXs from GBM cells overexpressing miR-7-5p (p < 0.05) (Figure 4B). These results suggested that VB could increase the expression of exosomal miR-7-5p.

Second, we speculated that the exosomes secreted by VB-stimulated GBM may be transferred to the tumor cells around it, according to our previous experimental results. So, we co-cultured U87 or U251 cells with the carboxyfluorescein succinimidyl ester (CFSE)-traced TEXs and observed the uptake of exosomes under a fluorescence microscope to verify whether the GBM cells can ingest the exosomes secreted by the same cells. These results showed that the uptake of exosomes by GBM cells was increased significantly over time (Figure 4C).

Meanwhile, the U87 or U251 cells were cultured with TEXs, which were extracted from GBM cells overexpressing miR-7-5p, after which, the expression of miR-7-5p in U87 or U251 cells was detected at 12, 24, and 48 h with qRT-PCR. The results revealed that the expression of miR-7-5p was increased over time (Figure 4D). These results suggested that TEXs of the tumor cells can transfer miR-7-5p to their recipient tumor cells.

In short, the expression of miR-7-5p in GBM was increased due to VB, and the miR-7-5p could be transferred to the recipient GBM cells by TEXs.

### Exosome containing miR-7-5p suppresses the proliferation, migration, invasion, and microtubule formation by inhibiting the activity of the EGFR/PI3K/Akt signaling pathway in GBM cells

**Figure 5:**
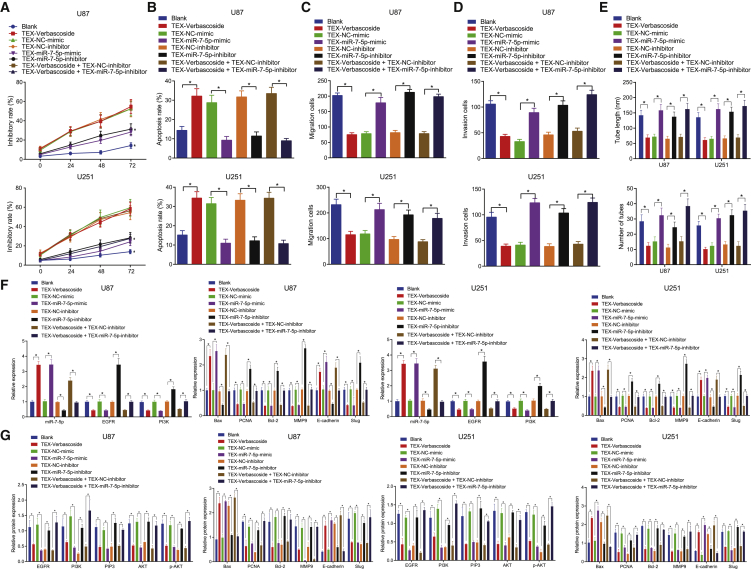
Exosome containing miR-7-5p inhibits the GBM cell proliferation, migration, invasion, and microtubule formation by inhibiting the EGFR/PI3K/Akt signaling pathway U87 and U251 cells were co-cultured with TEXs from VB-treated cells, from miR-7-5p-mimic-transfected cells, or from miR-7-5p-inhibitor-transfected cells. (A) Cell proliferation was detected by MTT assay. (B) Cell apoptosis was detected by flow cytometry. (C) Cell migration ability was detected by Transwell assay. (D) Cell invasion ability was detected by Transwell assay. (E) Angiogenesis of GBM cells was measured by microtubule formation. (F) The mRNA expression of related proteins (EGFR, PI3K, Bax, PCNA, Bcl-2, MMP9, E-cadherin, and Slug) was detected by qRT-PCR. (G) The expression of related proteins (EGFR, PI3K, PIP3, Akt, p-Akt, Bax, PCNA, Bcl-2, MMP9, E-cadherin, and Slug) was detected by western blot analysis. Data were shown as mean ± standard deviation of three technical replicates. ANOVA of repeated measurement was used for data comparison in (A). Unpaired t test was used for data comparison between two groups and one-way ANOVA for data comparison among multiple groups, with Tukey’s post-test test. ∗p < 0.05 compared with the corresponding control group.

We further verified whether miR-7-5p can be transmitted to the GBM cells through TEXs to subsequently exert its inhibitory effect and whether this secretion form can be produced by VB stimulation. We extracted TEXs from GBM cells transfected with miR-7-5p mimic or miR-7-5p inhibitor and TEXs from VB-treated cells for 48 h and then co-cultured U87 and U251 cells with the extracted TEXs. Next, the proliferation, apoptosis, invasion, migration, and microtubule formation of GBM cells were detected by a series of techniques including MTT assay, flow cytometry, and Transwell assay. The results showed an evident decline in the proliferation, invasion, migration, and microtubule formation ability in the cells co-cultured with the TEXs from VB-treated cells and an enhancement in the apoptosis, which indicated that the TEXs stimulated by VB could be transferred to GBM cells and produce anti-tumor effects. Additionally, the biological characteristics of GBM cells in the TEX-miR-7-5p-mimic group were significantly decreased, and the apoptosis rate was significantly enhanced in comparison with the TEX-negative control (NC)-mimic group, which was consistent with findings from the TEX-VB group; the proliferation, invasion, migration, and microtubule formation of GBM cells in the TEX-miR-7-5p-inhibitor group were significantly enhanced, and the level of apoptosis was significantly decreased in comparison with the TEX-NC-inhibitor group. Meanwhile, we cultured the U87 and U251 with the mixtures of TEXs after the action of VB and TEXs, which inhibited the expression of miR-7-5p, respectively. Our results showed that the biological characteristics of the TEX-VB plus TEX-miR-7-5p-inhibitor group were significantly enhanced, whereas the apoptosis rate was significantly reduced in comparison with the TEX-VB plus TEX-NC-inhibitor group, which identified that the anti-cancer effect of TEX-VB was annulled by the TEX-miR-7-5p inhibitor (p < 0.05) (Figures 5A−5E). The aforementioned results indicate that VB promotes the expression of miR-7-5p in GBM, and the miR-7-5p elicits its anti-tumor properties by exosomal delivery.

According to the paper’s reports, miR-7-5p can target EGFR and inhibit its expression,^18^ and the inhibition of the EGFR/PI3K/Akt signaling pathway can yield the anti-tumor effects on GBM cells.^19^ In this study, we detected the expression of miR-7-5p, proliferation- and apoptosis-related genes, and EGFR/PI3K/Akt signaling pathway-related genes by qRT-PCR and western blot analysis after extracting RNA and protein from GBM cells of each group. Our results showed that, in comparison with the blank and TEX-NC-mimic group, the expression of Bax and E-cadherin in the TEX-VB group and the TEX-miR-7-5p-mimic group was significantly higher, whereas that of EGFR, PI3K, PIP3, PCNA, Bcl-2, MMP9 and Slug, and Akt phosphorylation (p-Akt) level was significantly lower. Conversely, the TEX-miR-7-5p-inhibitor group was opposite to the TEX-VB group and TEX-miR-7-5p-mimic group when compared with the TEX-NC-inhibitor group (p < 0.05) (Figures 5F and 5G). These results suggested that VB could stimulate the secretion of exosomes by GBM cells to subsequently transfer miR-7-5p to other GBM cells and that VB could regulate the PI3K/AKT signaling pathway by targeting EGFR; meanwhile, the tumor-inhibiting role of VB could be counteracted by miR-7-5p inhibition.

To conclude, exosome-delivered miR-7-5p could inhibit cell proliferation, migration, invasion, and microtubule formation by inhibiting the EGFR/PI3K/Akt signaling pathway in GBM cells.

### VB promotes the delivery of miR-7-5p in the form of exosomes of GBM cells and inhibits the tumorigenesis and metastasis of GBM cells *in vivo*

**Figure 6:**
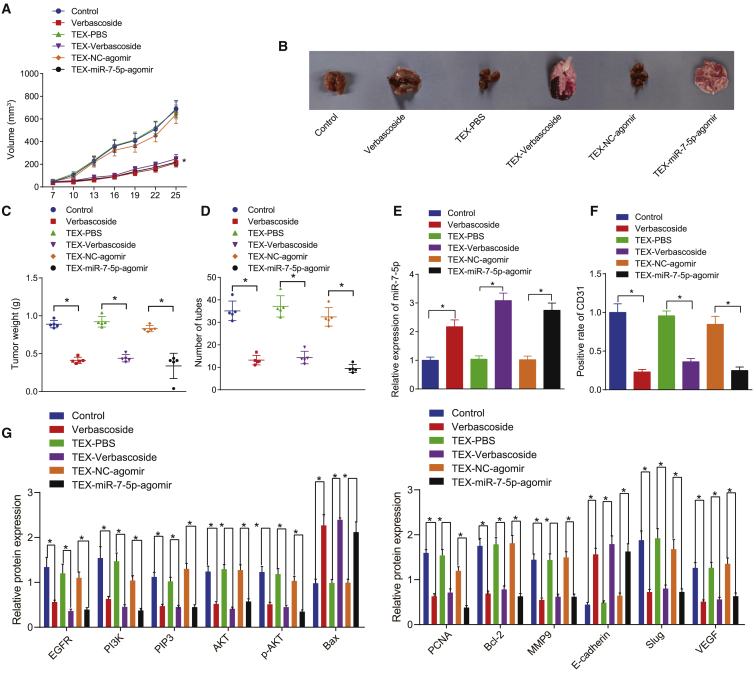
VB promotes the delivery of miR-7-5p by exosomes and inhibits the tumor formation and metastasis in mice Nude mice were treated with VB, TEX from VB-treated GBM cells, or TEXs from miR-7-5p-agomir-treated GBM cells. (A) Tumor volume was measured. (B) Representative images of tumorigenesis and lung metastasis in mice. (C) Statistical chart of tumor weight of mice. (D) The number of pulmonary metastases in mice. (E) The expression of miR-7-5p in tumor tissues was detected by qRT-PCR. (F) The positive expression of CD31 protein in tumor tissues was detected by immunohistochemistry. (G) The protein expression of EGFR, PI3K, PIP3, Akt, p-Akt, Bax, PCNA, Bcl-2, MMP9, E-cadherin, Slug, and VEGF in tumor tissues was detected by western blot analysis. Data were shown as mean ± standard deviation of three technical replicates. ANOVA of repeated measurement was used for data comparison in (A). Unpaired t test was used for data comparison between two groups and one-way ANOVA for data comparison among multiple groups, with Tukey’s post-test test. ∗p < 0.05, compared with the corresponding control group.

The subcutaneous tumor model and tumor metastasis model of nude mice were established by injecting U87 cells subcutaneously or via caudal vein, respectively, to ascertain that VB can promote the transfer of miR-7-5p through TEXs and inhibit the tumorigenesis and metastasis of GBM cells *in vivo*. We observed and recorded the formation of transplanted tumor in real time by injecting extracted TEXs via caudal vein or directly into VB, in which the TEXs were extracted from GBM cells treated with VB or transfected with miR-7-5p in each group. Our results showed that the tumor volume, weight, and number of pulmonary surface metastases were decreased significantly in the VB group, TEX-VB group, and TEX-miR-7-5p-agomir group in comparison with the corresponding saline control group, TEX-phosphate-buffered saline (PBS) group, and TEX-NC-agomir group (p < 0.05) (Figures 6A−6D).

After the experiment, the mice were euthanized. We harvested the subcutaneous transplanted tumors, fixed and embedded with paraffin and sections, after which, the expression of CD31 was detected in tumor tissues of each group by immunohistochemistry. Meanwhile, we detected the expression of miR-7-5p, EGFR/PI3K/Akt signaling pathway-related proteins (PI3K, PIP3, Akt, p-Akt, Bax, PCNA, Bcl-2, MMP9, E-cadherin, and Slug), and vascular endothelial growth factor (VEGF) by qRT-PCR and western blot analysis. The obtained results showed that the expression of miR-7-5p, E-cadherin, and Bax was significantly increased in the VB, TEX-VB, and TEX-miR-7-5p-agomir groups in comparison with the control, TEX-PBS, and TEX-NC-agomir groups, whereas that of the EGFR/PI3K/Akt signaling pathway-related genes, CD31 and VEGF, was decreased significantly (p < 0.05) (Figures 6E−6G). The results showed that the EGFR/PI3K/Akt signaling pathway was inhibited in the tumor, which considerably weakened the tumor angiogenesis ability.

Overall, VB might facilitate the delivery of miR-7-5p by exosomes derived from GBM cells and prevented the tumorigenesis and metastasis of GBM cells *in vivo*.

## Discussion

**Figure 7:**
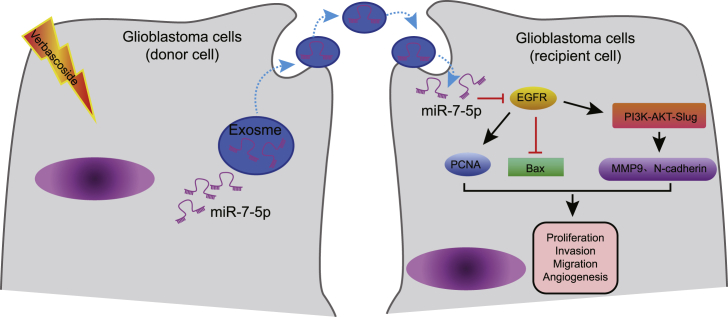
Mechanistic illumination of the inhibitory role of VB in GBM VB promotes the expression of miR-7-5p in GBM cells and facilitates the delivery of miR-7-5p by exosomes to recipient GBM cells. Then miR-7-5p inhibits GBM cell proliferation, migration, invasion, and microtubule formation *in vitro* as well as retarding the tumorigenesis and metastasis of GBM *in vivo* by inhibiting the EGFR/PI3K/Akt signaling pathway.

Due to the associated high morbidity and mortality with GBM,^21^ there is an urgent need to develop more effective treatment protocols. Recently, numerous studies have elicited the significance of traditional Chinese medicine in the treatment of various types of tumors. VB is one of the phenylpropanoid compounds from lemon verbena and has demonstrated a wide range of biological activities, including anti-inflammation, antioxidant, and immunomodulation.^22^ Moreover, previous research has shown that VB had a broad anti-cancer activity. For instance, Zhou et al.^23^ reported that VB promotes HIPK2-p53-mediated apoptosis in colorectal cancer cells. In addition, another study reported that VB inhibited lung cancer cell growth.^24^ Furthermore, a previously conducted study by Jia et al.^8^ highlighted that VB could inhibit the progression of GBM via downregulation of STAT3. In the current study, we tried to explore the therapeutic effect of VB on the GBM development corresponding with exosomal miR-7-5p from the GBM cells. Largely, our findings demonstrated that VB increased the expression of miR-7-5p in the GBM cells and delivered miR-7-5p via exosomes, thereby exhibiting an inhibitory effect on the development of GBM (Figure 7).

As one incurable cancer, the survival rate of GBM has not noticeably improved despite the advancements ascertained in new surgical and treatment protocols as well as improvements in radiation techniques.^25^ Accumulating evidence has attributed the increased treatment resistance and tumor recurrence to the proliferation of cancer cells and their interaction within the tumor microenvironment.^26^ VB significantly inhibits tumor cell metastasis and invasion by inhibiting Rac-1, hypoxia inducible factor-1α (HIF-1α), and Zeb-1 signaling pathways, which may be the most metastatic phenotype of colon cancer-suitable drug candidates.^27^ Similarly, it has been reported that VB exerted an inhibitory effect on the proliferation, migration, and invasion of GBM cells by enhancing the expression of protein tyrosine phosphatase SHP-1 and decreasing STAT3 phosphorylation.^8^ In this study, we also found that the supernatant of GBM cells stimulated by VB instead of VB itself could inhibit the vessel-like tube formation. Accordingly, VB has been demonstrated to repress GBM cell viability, invasion, migration, and tumor growth while promoting GBM cell apoptosis by increasing let-7g-5p expression and inhibiting high-mobility group (HMG)A2 expression.^28^ Our study findings demonstrated that miR-7-5p was poorly expressed in GBM, and the administration with VB induced the secretion of exosomal miR-7-5p, which could consequently exercise an inhibitory effect on GBM cell proliferation, invasion, migration, and vessel-like tube formation. Currently, miR-7-5p has been highlighted to function as a tumor suppressor involved in various cancers, including GBM. A recent report showed that miR-7-5p inhibited the migration and invasion of GBM cells by targeting special AT-rich sequence-binding proteins 1 (SATB1).^29^ Moreover, an association has been reported between the downregulation of miR-7-5p expression with the recurrence of patients with GBM.^30^ Essentially, existing evidence has established that the upregulation of miR-7-5p enhances the sensitivity of drug-resistant GBM cells to temozolomide (TMZ), a DNA-methylating agent, highlighting miR-7-5p as a promising therapeutic strategy to increase the long-term drug response in GBM patients.^30^ Moreover, miRNA microarray from a previous study also confirmed the presence of decreased expression of miR-7-5p in GBM patients and denoted that it may exert crucial functions in regulating glioma signaling pathways.^31^ Accumulating evidence confirmed that GBM cells could release microvesicles (exosomes), which possess the potential to induce tumor growth and provide valuable diagnostic biomarkers.^32^ miR-7-5p has been confirmed to be abundant in exosomes and can be delivered by exosomes to recipient cells and exert functions,^33^^,^^34^ which is in agreement with our results. Therefore, exosome-containing miR-7-5p as a result of VB administration may be a crucial mediator of GBM cell biological functions.

Furthermore, the present results revealed that exosomal miR-7-5p could inhibit GBM cell proliferation, invasion, migration, and vessel-like tube formation essentially by hindering the EGFR/PI3K/Akt signaling pathway. An existing study reported that EGFR and its downstream signaling pathways are vital to multiple cell processes, including cell proliferation and survival, in many tumors.^35^ Peculiarly, highly expressed EGFR has been found in the setting of GBM.^36^ Research has ascertained the involvement of suppression of the EGFR/PI3K/Akt/mTOR signaling pathway in the oxymatrine-mediated anti-tumor effects on GBM cells.^19^ Inhibition of the PI3K/Akt/mTOR signaling pathway has a promising clinical efficacy since activation of autophagy by the PI3K/Akt/mTOR inhibitors can enhance treatment sensitivity and trigger cell survival once drug resistance occurs in cancer cells.^37^ More importantly, miR-7-5p was verified to target EGFR, which has the capability to regulate gastric cancer cell proliferation and apoptosis through regulation of downstream pathways.^18^ Notably, reduction in the miR-7-5p expression contributes to the increase of EGFR expression and activation of the PI3K/AKT/c-Myc signaling pathway in the context of glioma.^38^

To conclude, our findings revealed that VB could suppress GBM cell proliferation, invasion, migration, and vessel-like tube formation. Additionally, VB could promote the expression of miR-7-5p in the GBM cells and transferred it to recipient GBM cells through exosomes. Besides, exosomal miR-7-5p could inhibit the progression of GBM *in vitro* and *in vivo* through blockade of the EGFR/PI3K/Akt signaling pathway. Our research confirmed for the first time that the exosomes produced by GBM cells after VB stimulation can regulate the biological function of GBM cells, thereby exerting the necessitated tumor-suppressive effects. These findings may provide new mechanistic insight into the pathogenesis of GBM and suggested a potential novel therapeutic target for GBM. On the other hand, the shortage of sufficient data from *in vivo* animal experiments is also noteworthy.

## Materials and methods

### Ethics statement

This study was approved by the Experimental Animal Ethics Committee and conducted in strict accordance with the recommendations in the *Guide for the Care and Use of Laboratory Animals* of the National Institutes of Health. Extensive efforts were made to ensure minimal suffering of the included animals.

### Cell culture

Human GBM cell lines U87^39^ and U251^40^ were purchased from the Cell Center of the Institute of Basic Medicine, Peking Union Medical College. These cells were cultured in an incubator under 37°C, 5% CO_2_, and saturated humidity using Roswell Park Memorial Institute (RPMI)-1640 medium (r8758; Gibco, Grand Island, NY, USA) containing 10% fetal bovine serum (FBS). The cells in the logarithmic growth period were collected, and the supernatant was removed by centrifugation after trypsin digestion. The cells were resuspended and adjusted to a concentration of 5.0 × 10^4^ cells/mL. VB was purchased from MedChemExpress (Monmouth Junction, NJ, USA) with purity of 99.61%. The powder was dissolved in dimethyl sulfoxide (DMSO) to prepare 1 mm stock solution. Then, 100 μL of cells was seeded into 96-well plates for 24 h and added with VB solution with variable concentrations of 0, 20, 40, 60, 80, and 100 μΜ, respectively. A blank control group containing only the culture medium and the NC group containing cells and PBS of the same concentration were set for this experiment. Each group repeated three times for each concentration.

### Cell transfection

The U87 and U251 cells in the logarithmic growth phase were digested using trypsin to prepare a single cell suspension, which was then seeded into 6-well plates. When reaching about 80% confluence, cells were transfected, according to the provided instructions of Lipofectamine 2000 reagents (11668-019; Invitrogen, Carlsbad, CA, USA) with vectors (NC-mimic, miR-7-5p-mimic, NC-inhibitor, and miR-7-5p-inhibitor; all purchased from Shanghai GenePharma, Shanghai, PR China). Each group’s miRNAs in 100 pmol were diluted using 250 μL of serum-free medium (Opti-minimum essential medium [MEM]), and 5 μL Lipofectamine 2000 was diluted by 250 μL serum-free medium (Opti-MEM), both of which were mixed and added to the 6-well plates after resting for 20 min. The final concentration of miRNAs added to cells was 50 nM. After transfection, the cells were cultured at 37°C with 5% CO_2_ and saturated humidity for 48 h. Then, the medium was replaced by RPMI-1640 medium for an additional 24−48 h of cell culture. Each experiment was repeated three times.

### Extraction and identification of TEXs

The GBM cells were cultured in FBS-free medium for 72 h. Then, the cell fragments and dead cells were removed by centrifuging at 1,200 × *g* and 4°C for 25 min. The supernatant was then filtered with a 0.2-mm filter, after which, the exosomes were isolated after ultracentrifugation at 120,000 × *g* and 4°C for 2.5 h. The exosomes were resuspended in PBS after a rinse with PBS and ultracentrifugation at 120,000 × *g* for 2 h. The extracted exosomes were used for the subsequent experiments.

The expression of exosome-specific surface markers CD63, TSG101, and CD9 was analyzed by western blot analysis so as to identify the characteristics of exosomes. The particle size of exosomes was determined using a Zetasizer Nano ZS (Malvern Instruments, Malvern, UK). The morphology of the TEXs was detected under a TEM (Tecnai Spirit; Fei, USA). Under the TEM, the exosomes were placed on Forvar and a carbon-coated copper grid, which was immersed on 2% phosphotungstic acid for 1 min and then rinsed with PBS.

### Uptake of CFSE-traced exosomes by GBM cells

CFSE dye (ab113853; Abcam, Cambridge, UK) was diluted at a ratio of 1:1,000, supplemented with 20 μg of the exosome suspension secreted from GBM cells after transfection, mixed, and left to stand for 15 min at 37°C. Next, the mixture was rinsed with PBS and centrifuged at 100,000 × *g* for 70 min. CFSE-traced exosomes were then co-cultured with GBM cells U87 for 12, 24, and 48 h. Finally, the uptake of exosomes by the GBM cells was visualized under a fluorescence microscope.

### Co-culture of GBM cell-derived exosomes and GBM cells

The exosomes were extracted from the GBM cells after transfection or VB treatment and then incubated with the GBM cells with the confluence of 60% in the 24-well plate for 48 h.^41^

All test groups were set as follows: the (1) blank group (cells treated with exosomes without VB treatment); (2) TEX-VB group (cells treated with exosomes derived from cells after 48 h of VB treatment); (3) TEX-NC-mimic group (cells treated with exosomes derived from NC-mimic-transfected cells); (4) TEX-miR-7-5p-mimic group (cells treated with exosomes derived from miR-7-5p-mimic-transfected cells); (5) TEX-NC-inhibitor group (cells treated with exosomes derived from NC-inhibitor-transfected cells); (6) TEX-miR-7-5p-inhibitor group (cells treated with exosomes derived from miR-7-5p-inhibitor-transfected cells); (7) TEX-VB plus TEX-NC-inhibitor group (cells treated with exosomes derived from VB-treated cells for 48 h and from NC-mimic-transfected cells); and (8) TEX-VB plus TEX-miR-7-5p-inhibitor group (cells treated with exosomes derived from VB-treated cells for 48 h and from miR-7-5p-inhibitor-transfected cells).

### MTT assay

After treatment with VB at different concentrations (0, 20, 40, 60, 80, and 100 μΜ) and transfection with different vectors, the U87 and U251 cells at the logarithmic growth phase in each group were seeded into 96-well plates at a density of 5 × 10^4^ cells/mL. Then 20 μL of MTT solution (5 g/L, sbj-0190; Nanjing, PR China) was added into each well after 24, 48, and 72 h.

After culture for 4 h, the medium was discarded, and each well was supplemented with 150 μL of DMSO and mixed by shaking for 10 min. The absorbance value of each well was measured by a SPECTROMAX i3x microplate reader (Meigu Molecular Instrument, Shanghai, PR China) to reflect cell growth viability. A cell growth curve was plotted based on the absorbance data. All experiments were repeated three times.

### Transwell assay

For cell-invasion experiments: Matrigel (YB356234; Shanghai Yubo Biotechnology, Shanghai, PR China) stored at −80°C was removed and liquefied overnight at 4°C. The upper chamber of the chamber bottom membrane was coated with a 1:8 diluent of Matrigel (200 mg/mL) (40111ES08; Yeasen Biotechnology, Shanghai, PR China). The chambers were dried overnight under sterile conditions. The cells were digested, counted, and prepared to suspension with the medium containing 20% FBS. The upper chamber was added with 5 × 10^4^ cells, whereas the lower chamber was added with 800 μL of medium containing 20% FBS and 50 μΜ of VB simultaneously. All cells were incubated in a 37°C incubator for 20−24 h. Transwell plates were rinsed twice with PBS, soaked in formaldehyde for 10 min, and then rinsed three times under running water. The cells were stained with 0.1% crystal violet, allowed to stand for 30 min at room temperature, and rinsed twice with PBS. Afterward, the cells on the surface were wiped off with cotton balls. Five visual fields were randomly selected for photography and documentation under an inverted microscope (XDS-800D; Shanghai Caikon Optical Instrument, Shanghai, PR China).

### Flow cytometric analysis

GBM cells seeded into the 6-well plates with different concentrations of VB (0, 20, 40, 60, 80, and 100 μΜ) and transfection were collected for this experiment according to the provided instructions of the Annexin V-fluorescein isothiocyanate/propidium iodide (FITC/PI) cell apoptosis detection kit (MA0220; Dalian Meilun Biotechnology, PR China). Briefly, cells were collected, rinsed with PBS, and counted. Then, the cells (2−5 × 10^5^ cells/mL) were collected and centrifuged at 500 × *g* for 5 min. The supernatant was then discarded, and the cells were resuspended with 195 μL of the binding buffer. Thereafter, 5 μL Annexin V-FITC was added to the cells and incubated in conditions devoid of light for 10 min at room temperature, followed by addition of 10 μL PI (20 μg/mL) and incubation in conditions devoid of light for 5 min at room temperature. All cells were detected by a flow cytometer. The blank control group, PI single-staining group, and Annexin V-FITC single-staining group were set in the experiment. The apoptosis of cells was finally measured using the FACSAria III system (BD Biosciences, Franklin Lakes, NJ, USA). The experiment was repeated three times.

### qRT-PCR

GBM cells seeded into 6-cm culture dishes with different concentrations of VB (0, 20, 40, 60, 80, and 100 μΜ) and transfection were collected for this experiment. Total RNA content was extracted from U87 cells, U251 cells, TEXs, and tumor tissues of nude mice using the TRIzol Extraction Kit (RP2401; BioTeke, Beijing, PR China). The primers for miR-7-5p, EGFR, and PI3K were designed and synthesized by Invitrogen with the sequences listed in Table 1. The extracted RNA was then reverse transcribed into complementary DNA (cDNA) using the PrimeScript RT Kit (RR036a; Takara). The volume of the reverse transcription system was 10 μL, with reference to the instructions. The reaction conditions were the following: 37°C, 3 times of reverse transcription reaction, 15 min for each, and 85°C for 5 s (RT inactivation reaction). Relevant primers were added to the ABI 7300 real-time fluorescent quantitative PCR instrument (Applied Biosystems, Foster City, CA, USA) for amplification and detection according to the instructions of the SYBR Premier Ex Taq II Kit (rr820a; Takara). The reaction conditions were set as follows: pre-denaturation at 95°C for 10 min, 40 cycles of denaturation at 95°C for 10 s, and annealing at 60°C for 20 s, as well as extension at 72°C for 34 s. The qRT-PCR reaction system was 20 μL: SYBR Premier Ex Taq II 10 μL, PCR forward primer (10 μM) 0.8 μL, PCR reverse primer (10 μM) 0.8 μL, ROX Reference Dye 0.4 μL, cDNA template 2.0 μL, and sterile distilled water 6.0 μL. The relative transcription level of miR-7-5p was calculated based on the relative quantitative method (2^−△△CT^), and 2 μg of the total RNA content was used as a template and U6 as an internal primer. The relative transcription level of EGFR and PI3K was calculated with glyceraldehyde 3-phosphate dehydrogenase (GAPDH) as the internal reference primer based on 2^−ΔΔCT^ (ΔΔCt = ΔCt_experiment group_ − ΔCt_normal group_, ΔCt = Ct_(target gene)_ − Ct_(internal reference)_). The relative transcription level of target gene mRNA = 2^−ΔΔCt^.^42^ Each experiment was repeated three times.

**Table 1:** Primer sequences for quantitative reverse transcriptase polymerase chain reaction

Target	Sequence (5′−3′)
miR-7-5p	forward: ACACTCCAGCTGGGTGGAAGA CTAGTGATTTT
	reverse: TGGTGTVGTGGAGTCG
U6	forward: CGCTTCGGCAGCACATATACT AAAATTGGAAC
	reverse: GCTTCACGAATTTGCGTGTCATCCTGC
EGFR	forward: AATGCGTGGACAAGTGCAAC
	reverse: CTCATTTCCTGGCCCGTAGG
PI3K	forward: AGCTGGTCTTCGTTTCCTGA
	reverse: GAAACTTTTTCCCACCACGA
GAPDH	forward: AATGGGCAGCCGTTAGGAAA
	reverse: GCGCCCAATACGACCAAATC

miR-7-5p, microRNA-7-5p; EGFR, epidermal growth factor receptor; PI3K, phosphatidylinositol 3-kinase; GAPDH, glyceraldehyde 3-phosphate dehydrogenase.

### Western blot analysis

GBM cells seeded into 6 cm culture dishes with different concentrations of VB (0, 20, 40, 60, 80, and 100 μΜ) and transfection, as well as tissue samples, were collected for this experiment. Total protein was extracted from tissues or cells with high-efficiency radioimmunoprecipitation assay (RIPA) lysis buffer (R0010; Beijing Solarbio Science & Technology, Beijing, PR China) in strict accordance with the provided instructions. The protein concentration of each supernatant was determined by a bicinchoninic acid (BCA) kit (20201ES76; Yeasen Biotechnology). The protein was separated by polyacrylamide gel electrophoresis. The protein was transferred onto the polyvinylidene fluoride (PVDF) membrane by the wet transfer method. The membrane was then blocked for 1 h at room temperature using 5% bovine serum albumin (BSA) and probed with the diluted primary rabbit antibodies against EGFR (1:1,000, ab131498; Abcam), PI3K (1:1,000, #4249; Cell Signaling Technology, Beverly, MA, USA), AKT (1:500, ab8805; Abcam), p-AKT (1:5,000, ab38449; Abcam), PCNA (1:1,000, ab18197; Abcam), MMP9 (1:1,000, ab73734; Abcam), Bax (1:1,000, ab53154; Abcam), Bcl-2 (1:1,000, ab59348; Abcam), E-cadherin (1:500, ab15148; Abcam), N-cadherin (1:1,000, ab18203; Abcam), Slug (1:500, ab27568; Abcam), VEGF (1:1,000, ab32152; Abcam), and GAPDH (1:2,500, ab9485; Abcam) at 4°C overnight. Following three washes using Tris-buffered saline-Tween 20 (TBST), 5 min each wash, the membrane was reprobed with goat anti-rabbit immunoglobulin G (IgG; 1:20,000, ab205718), labeled with horseradish peroxidase (HRP) for 1 h at room temperature. After development, the protein quantitative analysis was conducted using the ImageJ 1.48 u software (National Institutes of Health, Bethesda, MD, USA), after which, the ratio of gray value of each protein to that of internal reference β-actin was used for protein quantitative analysis. The experiment was repeated three times.

### Microtubule formation *in vitro*

The aim of this experiment is to observe the effect of GBM cells following different treatments on the formation of microtubules in the HUVECs (354151; Corning, Corning, NY, USA). HUVECs and GBM cells were cultured with DMEM containing 10% FBS and RPMI-1640 medium, respectively, in a 37°C incubator. The cells were divided into the NC group (treated with PBS, exosomes following PBS addition, or exosomes from cells transfected with negative plasmids) and the experimental group (treated with VB, exosomes following VB addition, or exosomes from cells transfected with the plasmid containing the target fragment). The supernatant of cells was collected for 48 h later, and then, the cell fragments were removed by aseptic centrifugation. At last, the supernatant of the tumor cell culture was obtained, and the tumor conditioned medium was prepared according to the ratio of 4 (tumor supernatant):5 (DMEM):1 (FBS). Each well of 96-well plates was added with 50 μL Matrigel and reacted in a 37°C incubator for 30 min. After coagulation, an appropriate amount of the amount of prepared tumor conditioned medium and HUVEC suspension was added to the plates, cultured at 37°C and 5% CO_2_ for 8 h. Each group was measured in triplicate. Four randomly selected fields were chosen, and the length and node number of microtubules were counted and photographed using a phase-contrast microscope.

### Animal experiment

Thirty specific pathogen-free (SPF)-grade female BALB/c nude mice (6 weeks, 15−18 g, purchased from SLAC Laboratory Animal) were randomly divided into six groups, five mice in each group. Then 0.2 mL single-cell suspension (including 1 × 10^6^ cells) of U87 GBM cells (concentration of 5 × 10^6^ cells/mL) was injected into the mice subcutaneously to construct a subcutaneous tumor model. The exosomes were extracted from U87 cells that had been transfected with miR-7-5p or treated with 50 μΜ of VB for 48 h. The control PBS or TEXs or VB was injected into the tail vein of the nude mice on the 5th, 10th, 15th, 20th, and 25th day, respectively. The specific groups were as follows: the (1) control group (mice were injected with PBS via tail vein); (2) VB group (mice were injected with 50 μΜ of VB via tail vein); (3) TEX-PBS group (mice were injected with TEXs from PBS-treated cells via tail vein); (4) TEX-VB group (mice were injected with TEXs from 50 μΜ of VB-treated cells for 48 h via tail vein); (5) TEX-NC-agomir group (mice were injected with TEXs from miR-187 empty vector-transfected cells via tail vein); and (6) TEX-miR-7-5p-agomir group (mice were injected with TEXs from miR-7-5p-transfected cells via tail vein). The volume of transplanted tumor for nude mice was measured and recorded in real time. The volume of tumor was calculated using the formula: V = A × B^2^/2 (mm^3^), in which A indicates the largest diameter, and B indicates the vertical diameter. On the 28th day, the mice were euthanized, with the transplanted tumor harvested and weighed. The extracted RNA and protein content from the transplanted tumor tissues were analyzed by qRT-PCR and western blot analysis. The tumor tissues were paraffin embedded and sectioned for histopathological analysis.

For tumor metastasis *in vivo*, 15 SPF female thymus-free BALB/c nude mice (6 weeks, 15−18 g, purchased from SLAC Laboratory Animal) were divided into 3 groups randomly, 5 mice in each group. The mice were injected with 2 × 10^6^ U87 cells treated with the aforementioned exosomes or VB via tail vein. 8 weeks after the injection, the mice were euthanized, all of the lungs were harvested, and the number of metastatic nodules was estimated.

### Immunohistochemistry

The paraffin tissue sections in each group were collected and used for immunohistochemistry analysis. Briefly, the sections were dewaxed, dehydrated with gradient alcohol, rinsed under tap water, and subjected to antigen retrieval in a water bath. Then, the sections were added with normal goat serum-blocking solution (C-0005; Shanghai Haoran Biotechnology, Shanghai, PR China) and then immunostained with the primary antibody against CD31 (1:50, ab28364) and secondary antibody against goat anti-rabbit IgG. Following three rinses using PBS (5 min for each), the sections were dropped with streptomyces ovalbumin working solution labeled with HRP (0343-10000U; Yimo Biotechnology, Beijing, PR China) and developed with 3,3′-diaminobenzidine tetrahydrochloride (DAB) (ST033; Guangzhou Weijia Technology, Guangzhou, PR China). Hematoxylin (PT001; Shanghai Bogu Biotechnology, Shanghai, PR China) was added to the sections for counterstaining. At last, the sections were observed under a microscope in 5 randomly selected high-power fields, with 100 cells counted for each field. The positive cells <10% were suggestive of negative, the positive cells ≥10% and <50% were positive, and the positive cells >50% were strong positive.

### Statistical analysis

All statistical analyses were conducted using SPSS 21.0 software (IBM, Armonk, NY, USA), with two-tailed p <0.05 as a level of statistical significance. Measurement data were presented as mean ± standard deviation from at least three independent experiments. Within-group data following normal distribution and homogeneity of variance were compared using paired t test or unpaired t test, whereas multigroup data at different time points were compared by one-way analysis of variance (ANOVA) or the ANOVA of repeated measurement, followed by Tukey’s post hoc test with corrections for multiple comparisons. Rank sum test was used for data with skewed distribution and defect variances. Counting data were analyzed by chi-square test. p <0.05 or p <0.01 was indicative of a significantly statistical difference.
